# Ecofriendly Synthesis and Insecticidal Application of Copper Nanoparticles against the Storage Pest *Tribolium castaneum*

**DOI:** 10.3390/nano10030587

**Published:** 2020-03-23

**Authors:** Mohamed T. El-Saadony, Mohamed E. Abd El-Hack, Ayman E. Taha, Moustafa M. G. Fouda, Jamaan S. Ajarem, Saleh N. Maodaa, Ahmed A. Allam, Nashwa Elshaer

**Affiliations:** 1 Department of Agricultural Microbiology, Faculty of Agriculture, Zagazig University, Zagazig 44511, Egypt; 2 Department of Poultry, Faculty of Agriculture, Zagazig University, Zagazig 44511, Egypt; 3 Department of Animal Husbandry and Animal Wealth Development, Faculty of Veterinary Medicine, Alexandria University, Edfina 22758, Egypt; 4 Pretreatment and Finishing of Cellulosic-based Fibers Department, Textile Industries Research Division, National Research Centre, 33 El-Buhouth Street, Dokki, Cairo 12622, Egypt; drmmfouda@gmail.com; 5 Department of Zoology, College of Science, King Saud University, P.O. Box 2455, Riyadh 11451, Saudi Arabia; jajarem@KSU.EDU.SA (J.S.A.); maodaa_28@yahoo.com (S.N.M.); 6 Department of Zoology, Faculty of Science, Beni-suef University, Beni-suef 65211, Egypt; allam1081981@yahoo.com; 7 Department of Plant Protection, Faculty of Agriculture, Zagazig University, Zagazig 44511, Egypt; nashwa.elshaer@yahoo.com

**Keywords:** copper nanoparticles, *Pseudomonas fluorescens*, insecticidal, *Tribolium castaneum*

## Abstract

In spite of great developments in the agricultural field and plant productivity in the last decades, the concern about the control of agricultural pests is still continuous. However, pest management is expected to have more effects from nanomaterials by providing innovative solutions. The current study confirms the biotransformation of copper nanoparticles (CuNPs) using a cell-free culture extract of metal copper-resistant bacteria *Pseudomonas fluorescens* MAL2, which was isolated from heavy metal-contaminated soils collected from Sharqia Governorate, Egypt. The local screened bacterial isolate, *Pseudomonas fluorescens* MAL2, is similar to *Pseudomonas fluorescens* DSM 12442T DSM. After optimization of growth conditions, F-Base medium was found to be the best medium and pH 7, temperature 35 °C, concentration of CuSO_4_·5H_2_O 300 ppm, 10 mL supernatant: 40 mL CuSO_4_·5H_2_O (300 ppm), and reaction time 90 min were recorded as the best growth conditions to the fabrication of CuNPs. The formed CuNPs were characterized using initially visual observation of the change in the color of the reaction mixture from blue color to the dark green as an indication of CuNPs biotransformation. Then, UV–Vis spectroscopy showed a maximum absorption at 610 nm under the optimum conditions performed. Transmission Electron Microscopy (TEM) revealed the formation of spherical aspect with size ranges from 10:70 nm; moreover, Energy Dispersive X-ray spectroscopy (EDX) indicated the presence of CuNPs and other elements. In addition, the presence of alcohols, phenols, alkenes, and amines is confirmed by Fourier-Transform Infrared spectroscopy (FTIR) spectroscopy analysis. Dynamic Light Scattering (DLS) supported that the Zeta-average size of nanoparticle was 48.07 with 0.227 PdI value. The Zeta potential showed −26.00mV with a single peak. The biosynthesized CuNPs (Bio CuNPs) showed toxicity against the stored grain pest (*Tribolium castaneum*), where LC_50_ value was 37 ppm after 5 days of treatment. However, the negligible effect was observed with chemical synthesis of CuNPs (Ch CuNPs) at the same concentration. The results suggest that Bio CuNPs could be used not only as a biocontrol agent, but also as an ecofriendly and inexpensive approach for controlling the stored grain pests.

## 1. Introduction

Over the past decade, many exploratory experiments have been conducted to demonstrate the effectiveness of the powerful impact of nanotechnology and its applications [1,2]. The size of solid nanoparticles is ranging from 1 to 100 nm [3,4,5]. Copper nanoparticles (CuNPs) have been used in full applications such as agriculture, medicine, environment, industrial engineering, and various technological fields. Recently, studies in the agriculture field emphasized the influence of some simple elements related to the economics of plants [4,5,6,7]. The combination of biological principles with different chemical and physical methods is called bio-nanotechnology producing nanoparticles with specific functions [8,9]. The chemical synthesis of nanoparticles is a very costly method, and it is considered toxic with low productivity. Subsequently, the biological synthesis methods from plants or microbes have attractive application because they are costless, safe, rapid, and ecofriendly [8,9,10].

The biotransformation of NPs using microbes has attracted much researcher interest because of its intrinsic qualities and potential applications in different fields. Each microorganism has a specific mechanism to form nanoparticles [11]. Target ions from the environment were grasped on the surface or inside the microbial cells, and then the cell enzymes can reduce the metal ions size to nanoparticles by the electrostatic interaction [12]. CuO NPs can be synthesized from Gram-negative bacterium, which belongs to the genus Serratia [13]. Non-pathogenic *Pseudomonas stutzeri* can produce spherical CuNPs with size ranges from 8 to 15 nm; however, it can synthesize cubical CuNPs sized 50 to 150 nm from electroplating wastewater [14]. Furthermore, *Escherichia coli* can synthesize CuO NPs with variable sizes and shapes [15]. Currently, there is an increasing interest in using nanoscience in agriculture for several purposes such as enhancing nutrient absorption and delivery, detecting diseases, and improving the pesticides deliveries to the target sites; thereby, it can enhance our understanding of the crop’s biology [16,17]. 

The essential role of nanoparticles for the control of agricultural arthropods and medical-veterinary pests was reported [18]. The arthropods represent the most abundant phylum of the animal kingdom with fundamental economic importance. Arthropods which include phytophagous insects and mite species can destroy not only the grown crops but also the stored agricultural products [19]. The red flour beetle, *Tribolium castaneum* Herbst (Coleoptera: *Tenebrionidae*), is a global stored product pest particularly stored in grains and foodstuffs [20], damaging their quality and quantity [21]. This beetle causes a significant lack in the weight and nutritive contents of grains, reducing the commercial value and reducing the germination ratio of grains [22]. *T. castaneum* has been globally used as a model organism in pesticide and ecotoxicology researches [23]. Copper nanoparticles (CuNPs) exhibited fungicidal and insecticidal activity against the pests of crop plants. They can be used as CuNP-based nano-pesticides, nano-herbicides, and nano-fertilizers [24].

Therefore, the current investigation describes the biotransformation of CuNPs using metal copper-resistant *Pseudomonas fluorescens* under optimized growth conditions, and it sheds lights on the characterization of the produced CuNPs by available advanced techniques, as well as investigating the CuNPs bioresources and their potential pesticidal activity against the storage pest (*T. castaneum*). 

## 2. Materials and Methods

Metal contaminated soil samples were collected from different sites next to some metal factories in Sharqia Governorate, Egypt. They were mixed and considered to be source material for bacterial isolation. The isolation of metal (Cu)-resistant bacteria was done by Replica plate technique using nutrient agar (NA) plates supplemented with 100 ppm of filtered and sterilized CuSO_4_·5H_2_O solution incubated at 37 °C for 48 h, and they were observed for the presence of bacterial growth [25], then the recovered cultured isolates were considered as copper-resistant bacteria. Only three bacterial isolates were recovered because of their good aspects and distribution on the plates. The bacterial isolates (MAL1, MAL2, and MAL3) were subjected to a screening process, which was done to choose the best isolate that biosynthesizes copper nanoparticles.

### 2.1. Screening for Copper Nanoparticles Synthesis

All the metal (Cu)-resistant bacterial isolates were further studied qualitatively for copper nanoparticle synthesis [25,26]. The cultures were cultivated separately at pH 7 and 37 °C for 24 h in Luria–Bertani broth (LB). Bacterial cells were separated by centrifugation at 20,000 rpm for 10 min. Cell-free filtrate material was separated out and used for extracellular synthesis of nanoparticles. For nanoparticle synthesis, approximately 10 mL of supernatant from each cell-free culture was mixed individually with 40 mL of 100 ppm of filtered and sterilized CuSO_4_·5H_2_O solutions (1:4 ratio) in 250 mL conical flasks containing 100 mL of the reaction mixture and placed in a 120 rpm incubatory shaker (LAB—Line R ORBIT environ shaker, AK, USA) at 37 °C up to 24 h. Qualitative screening for the presence of copper nanoparticles was done by periodic visual observation to check the color change, where the color changed from blue to heavy green. The appearance of heavy or dark green color in the flask reveals the biotransformation of CuNPs in the reaction mixture [27], also a set of flasks without CuSO_4_·5H_2_O were maintained as control. 

### 2.2. Bacterial Isolates Identification

The chosen isolate was identified as per their morphological, biochemical, and physiological characteristics through tests designated in Manual of Systematic Bacteriology by Bergey’s et al. [28]. It was then identified at molecular level by matrix-assisted laser desorption ionization-time of flight (MALDI TOF, VITEK MS RUO; bioMérieux, Marcy l’Etoile, France) mass spectrometry [29], to confirm the previous identity of isolated bacteria.

### 2.3. Biosynthesis of CuNPs by Pseudomonas fluorescens

The isolated bacteria, *Pseudomonas fluorescens* MAL2, was grown in F-Base medium flasks (Kings B medium), which contained peptone, 20 g/L; K_2_HPO_4_, 1.5 g/L; MgSO_4_, 1.5 g/L; Agar, 15 g/L and glycerol, 10 mL pH 7.2, where the flasks were incubated at 30 °C and 150 rpm for incubation (Lab-line^®^ Incubator-Shaker-ORBIT-USA, Suite D Annapolis Junction, MD 20701, USA). Just after 48 h, the culture was subjected to centrifugation at 10,000 rpm for 20 min at 30 °C for cell pellet and cell-free extract collection [27].

One gram of cell pellet was suspended separately in 20 mL of (100, 300, 500, 700 and 900 ppm) CuSO_4_·5H_2_O solutions. Some altered volumes (10, 20, 10, 40, 40 and 50mL) of cell-free supernatant were also added to several concentrations (100, 300, 500, 700, and 900 ppm) of CuSO_4_·5H_2_O solutions in the prepared conical flasks. Then, all flasks were set in shaker incubator for 24 to 48 h at 35 °C and 150 rpm, and then they were investigated for CuNPs biotransformation by using UV–Vis analysis (400–800 nm “model Laxco™, Alpha-1502 Alpha Series Spectrophotometer (Mettler-Toledo, LLC 1900 Polaris Parkway Columbus, OH 43240S)”, 200–1000 nm [30].

### 2.4. Optimization of CuNPs Biotransformation

Six different parameters, i.e., media type, CuSO_4_·5H_2_O concentration, different volumes of cell-free supernatant, pH values, reaction time, and temperature degrees were tested to attain the maximum growth conditions for the biosynthesis of CuNPs, where only one factor was variable, while the other factors were kept constant. *Pseudomonas fluorescens* MAL2 inoculum (2.5 × 10^6^ CFU/ mL) was prepared and inoculated in three different media flasks): Nutrient broth (NB), F-Base media (Kings B media), and Luria–Bertani broth (LBB), and then incubated at 30 °C and 150 rpm for 48 h. Different concentrations of CuSO_4_·5H_2_O (100, 300, 500, 700, and 900 ppm) were added to prepared cell-free supernatants. After this, the optimum concentration recorded from (CuSO_4_·5H_2_O) solution was added to the cell-free supernatants at different pH values (4, 5, 6, 7, 8, and 9), and then incubated at 30 °C. The recorded values from the previously testes parameters were used to determine the mixing ratios of cell-free supernatant to CuSO_4_·5H_2_O (10:50, 20:40, 10:40, 40:10, 40:20, and 50:10). Finally, the reaction time (30, 60, 90, 150, and 180 min) was also studied using the optimized different parameters. The obtained mixture reactions samples were analyzed at the appropriate times using UV–Vis spectral analysis (400–800 nm model “Laxco™, Alpha-1502 Alpha Series (Mettler-Toledo, LLC 1900 Polaris Parkway Columbus, OH 43240)” Spectrophotometer, 200–1000 nm [30]. After optimization of the measured parameters, the recorded values were applied collectively in an experiment to yield CuNPs, separation and purification processes.

### 2.5. Characterization of Copper Nanoparticles

Initially, CuNPs formation was recorded by visual observation of color change by the naked eye. Then, the formed CuNPs were again characterized by UV–Vis spectroscopy, prepared mixture reaction was monitored using Laxco™ dual-beam spectrophotometer (Mettler-Toledo, LLC 1900 Polaris Parkway Columbus, OH 43240), model Laxco™, Alpha-1502 Alpha Series Spectrophotometer (Mettler-Toledo, OH 43240), 200–1000 nm) [30] for detecting surface plasmon resonance (SPR) of the obtained absorbance peak for CuNPs. Fourier Transform Infrared Spectroscopy (FTIR) analysis was performed as per [31] (Bruker Tensor 37, Kaller, Germany). Moreover, the suspension of CuNPs was dried [32] and exposed to EDX using a JEOL MODEL JSM-IT200 Series (Peabody, MA 01960, USA) [33]. The morphology and size of CuNPs were determined by TEM. To this end, an aliquot of an aqueous suspension of CuNPs was transferred onto an amorphous carbon-coated copper grid, left for drying, and investigated using TEM JEOL 1010, Japan, as per [34]. A Zetasizer analyzer (Nano “Z2 Malven, Malvern Hills, UK”), for the analysis of Zeta potential, was employed as per the work in [35].

### 2.6. Chemical Synthesis of CuNPs

The copper nanoparticles were synthesized by using a chemical reduction method described in [36].

### 2.7. Insect Rearing

The red flour beetles, *T. castaneum*, were reared on ground wheat grains mixed with 5% dried yeast under laboratory conditions 32 ± 2 °C and 68 ± 2% rh., and 12:12 h (Light: Dark). Beetle adults used in the bioassay experiment were 7–14 days old.

### 2.8. Insect Bioassay

Efficacy of chemical and biosynthesis of CuNPs against *T. castaneum* was tested at six different concentrations (300, 250, 200, 150, 100, and 50 μg mL^−1^), which were prepared by dilutions with distilled water. Distilled water was used as a negative control treatment. Procedures materials used in the biosynthesis of copper nanoparticles (*Pseudomonas* supernatant and CuSO_4_·5H_2_O with 300 ppm concentration) were evaluated as a positive control. Two milliliters of each concentration were added carefully to 20 g of wheat grains and shaken to uniformly distribute the used concentration on wheat grains, then left to air dry. Twenty adults were exposed to each concentration separately. Three replicates were performed in all experiments. The mortality rate of adults was registered after 24 h till the fifth day.

## 3. Statistical Analysis

Probit analysis, according to the work in [37], using IBM SPSS software (Armonk, NY 10504-1722, USA, version 20) was employed in analyzing the concentration-mortality response. LC_50_ values for each exposure period and their fiducial limits were estimated. Control mortality was zero, and no corrections were necessary.

## 4. Results and Discussion

### 4.1. Isolation of Metal Resistant Bacteria from Soil

From the isolation process, it was observed that among the cultured colonies, only three isolates were recovered on the basis of their good growth and aspect on nutrient agar plates supplemented with 100 ppm concentration of CuSO_4_·5H_2_O. Therefore, they were considered as copper-resistant bacterial isolates, and they have the ability to biosynthesis the CuNPs. Findings on the existence of copper-resistant bacteria in copper-contaminated soils are in accordance with Altimira et al. [38], as they have isolated the silver-resistant bacteria from heavy metal contaminated areas. The copper-resistant soil isolates, showing the growth in the presence of CuSO_4_·5H_2_O, were stained with Gram’s stain. The results confirmed the presence of similar morphological forms such as short bacilli with a variety of arrangements and three species of Gram-negative bacilli were observed and slanted. 

### 4.2. Screening for Copper Nanoparticles Synthesis

**Figure 1:**
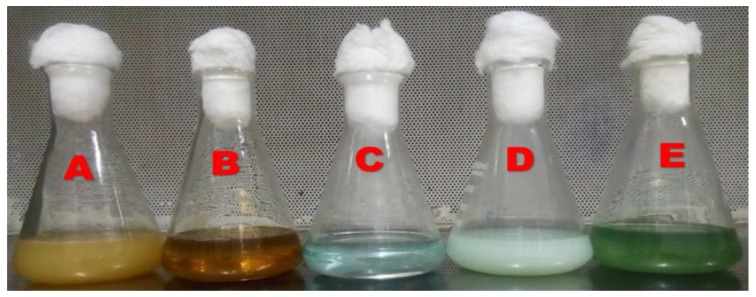
Observation of the color change in the reaction mixture of *Pseudomonas fluorescens* MAL2 during the biosynthesis of CuNPs: (**A**) *P. fluorescens* MAL2 in F-Base medium. (**B**) Cell-free extract supernatant. (**C**) CuSO_4_·5H_2_O solution. (**D**) One gram of cell pellet added to CuSO_4_·5H_2_O. (**E**) Color change in the reaction mixture from blue to dark green indicating the formation of CuNPs.

Cell-free supernatants of all copper-resistant bacterial isolates were separately treated with 100 ppm of filtered and sterilized CuSO_4_·5H_2_O solutions. After the incubation time, the reaction mixtures in the growing flasks were observed for color change from blue to dark green (Figure 1). Only one isolate out of three clearly showed the synthesis of CuNPs. The second isolate showed the synthesis of CuNPs quickly within one hour, whereas the first and the third did not have the ability to synthesize CuNPs. These findings are in agreement with the experimental findings of Zaki et al. [39]. The aforementioned authors reported that the color change from blue to dark green is due to the biosynthesis of CuNPs. From the results of the qualitative screening of CuNPs producer, it was also sensible that the character to have copper resistant traits among the strains must not be taken as the only final decision or indicator for the biotransformation of CuNPs. The qualitative screened isolate was nominated as *Pseudomonas fluorescens* MAL2 as CuNPs producer and subjected to molecular identification.

### 4.3. Identification of the Screened Bacteria

The screened microbe was Gram-negative, motile, short rod, and non-spore-forming under a light microscope, and aerobic in its oxygen requirements. Regarding the morphological results and biological tests that were conducted on the selected microbe as per protocols recommended in Bergey’s Manual [40] and comparison process, which was held in this respect, it can be inferred that the local isolate was *Pseudomonas fluorescens*, and it was denoted as *Pseudomonas fluorescens* MAL2. Then, it was further exposed again to fast and precise identification by MALDI TOF Mass spectrometry (VITEK MS RUO; bioMérieux, Marcy l’Etoile, France). The registered results observed its maximum similarity of 98% to several *Pseudomonas* spp. predominantly *Pseudomonas fluorescens* DSM 12442T DSM [38]. Thus, the local screened bacterial isolate (*Pseudomonas fluorescens* MAL2) is similar to *Pseudomonas fluorescens* DSM 12442T DSM.

### 4.4. Optimization Factors

#### 4.4.1. Medium Type

**Figure 2:**
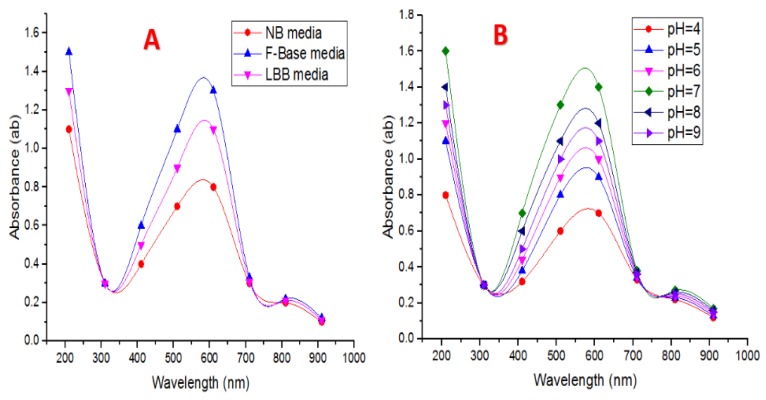
(**A**) UV–Vis spectra of medium type. (**B**) UV–Vis spectra of different pH values utilized throughout CuNPs biotransformation by *P. fluorescens* MAL2.

Three different media—Nutrient broth (NB), F-Base broth (Kings B medium), and Luria–Bertani broth (LBB)—were utilized to explore the best medium type in this experiment. UV–Vis spectra of CuNPs produced by the tested bacteria after incubation and growth in three different media are presented in Figure 2A. The absorbance in the case of NB was 0.81 nm, whereas it was 1.43 nm in the case of F-Base medium and this absorbance in LBB was 1.19 nm at 650 nm. The F-Base medium was found to be the best medium for the fabrication of CuNPs using the tested *Pseudomonas fluorescens* MAL2 bacteria. As for medium type, F-Base medium was found to be the best medium for the fabrication of CuNPs by *Pseudomonas fluorescens* MTCC 103 [41]. 

#### 4.4.2. pH Levels 

It is well-known that the change in the pH values can control the shape and size of NPs [42]. Peaks recorded at acidic pH of four, five, and six in the media revealed that low pH decreased the biotransformation of CuNPs. Low pH did not favor CuNPs biotransformation (Figure 2B). Alkaline pH presented a sharp peak at 561 nm, indicating the presence of larger-sized particles or aggregation. Increase or decrease of alkalinity can lead to aggregation or alteration of nanoparticles which was likewise found in *E. coli*. In this experiment, at pH 7, a characteristic peak observed at 610 nm proves CuNP fabrication. Figure 2B reveals that pH 7 is the best for the NPs fabrication. Gold nanoparticles are biotransformed at pH 7 using *Rhodopseudomonas capsulate*, as stated earlier by He et al. [43]. Shantkriti and Rani [27] reported that pH seven was found to be the optimum pH for the production of CuNPs by *Pseudomonas fluorescens* MTCC 103.

#### 4.4.3. Temperature Degrees

**Figure 3:**
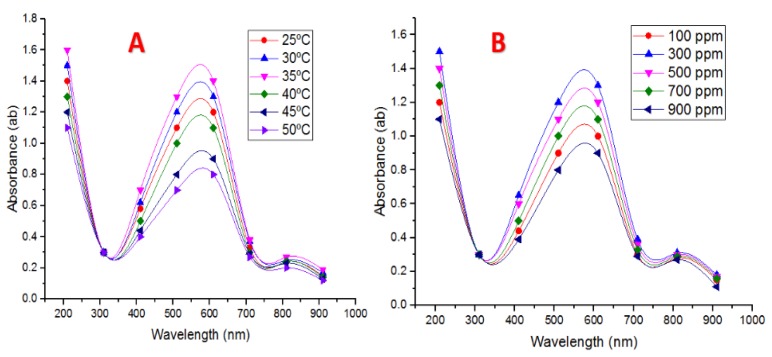
(**A**) UV–Vis spectra of different temperatures utilized. (**B**) UV–Vis spectra of CuSO_4_·5H_2_O concentration used during CuNPs biotransformation by *P. fluorescens* MAL2.

Six different temperature degrees were performed for studying this factor, i.e., 25, 30, 35, 40, 45, and 50 °C. It was easy to see that 35 °C was recorded to be the optimum temperature for the biotransformation of CuNPs by the tested *Pseudomonas fluorescens* MAL2 bacteria (Figure 3A). The obtained results proved that the increase in temperature reduced the production of CuNPs, and this reduction could have occurred because of the inactivation or degradation of biocompounds in the reaction mixture responsible for the bioreduction process. As the reaction temperature increased in the reaction mixture, both synthesis rate and conversion to copper nanoparticles in the medium increased [44].

#### 4.4.4. CuSO_4_·5H_2_O Concentrations and Supernatant on CuNPs Biotransformation

To study the influence of CuSO_4_·5H_2_O concentration, five concentrations of CuSO_4_·5H_2_O (100, 300, 500, 700, and 900 ppm Cu) were utilized as revealed in Figure 3B. Control samples did not show any distinct peak in the range of 550 to 650 nm and indicated no formation of CuNPs. As the cell-free extract concentration was increased in the reaction mixture to 700 and 900 ppm Cu, clear absorption peaks were perceived in the region above 700 nm. When 10 mL of cell-free extract was added to 300 ppm CuSO_4_·5H_2_O solution, a specific peak was detected; suggesting 10 mL supernatant was sufficient to reduce copper ions in mixture reaction to form CuNPs. A further increase in the volume of cell-free extract (40 mL) to 900 ppm copper solution leads to peak sharpening with the aggregation of CuNPs. This result is in accordance with Shantkriti and Rani [27], as they found that when 10 mL of a cell-free extract of *Pseudomonas fluorescens* was added to 318.4 ppm copper sulfate solution, a characteristic peak was recorded, suggesting that 10 mL cell-free extract was adequate to decrease Cu^+2^ ions in solution to form CuNPs. 

#### 4.4.5. Mixing Ratio of Cell-Free Extract and CuSO_4_·5H_2_O 

**Figure 4:**
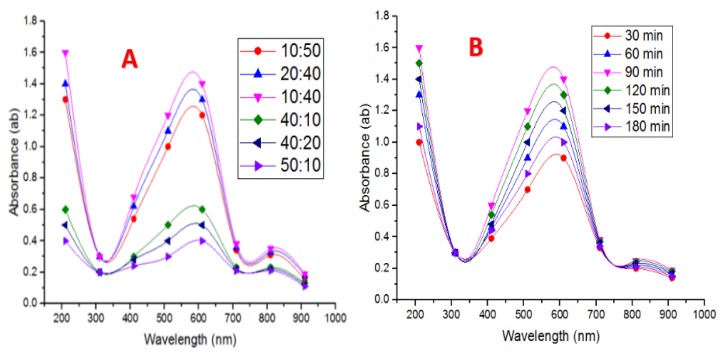
(**A**) UV–Vis spectra of mixing relation of culture supernatant and CuSO_4_·5H_2_O. (**B**) UV–Vis spectra of incubation times used during CuNPs biofabrication by *P. fluorescens* MAL2.

Six different volumes from 300 ppm CuSO_4_·5H_2_O as stock solution were mixed with six different volumes of a cell-free extract of *Pseudomonas fluorescens* MAL2 (10:50, 20:40, 10:40, 40:10, 40:20, and 50:10, respectively) and incubated for 24 h at 35 °C. UV–Vis spectra of CuNPs were recorded, and are given in (Figure 4A). The results showed that volumes of 10 mL of 300 ppm CuSO_4_·5H_2_O, which were added with 40 mL of cell-free extract at 1: 4 ratios, were deemed to be the best ratio. As it provided final absorbance at 610 nm, the increment in cell-free extracts volumes caused a reduction in the absorbance. This decline proved a reduction in NPs size. This indicates that by increasing the amount of cell-free extract in the reaction mixture, the amount and size of NPs become smaller [45]. 

#### 4.4.6. Reaction Time on Nanoparticle Production

Time is an important feature that enhances NPs biotransformation and stability. From the reaction time experiment performed (30, 60, 90, 120, 150, and 180 min), it was seen that the absorbance of the reaction mixtures at 610 nm increased gradually up to 90 min, and then it started to decrease (Figure 4B). This trend suggests that CuNP biotransformation occurs, with an increase in reaction time; size reduction takes place, and CuNP biotransformation was performed under optimum conditions. To clarify the reaction time process, the UV–Vis absorption spectra were registered every 10 min [46]. 

### 4.5. Characterization of Copper Nanoparticles

#### 4.5.1. UV–Vis Analysis of CuNPs Biosynthesis

**Figure 5:**
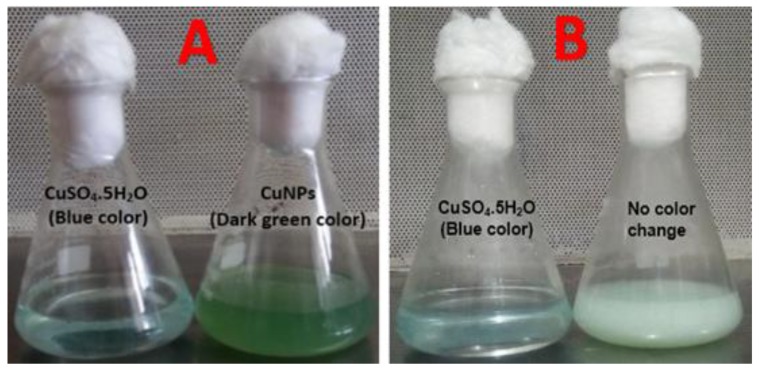
(**A**) Color change in the in the intensity of the reaction of CuNPs formation. (**B**) No color change occurs when 1 g of cell pellet is added to the CuSO_4_·5H_2_O solution.

**Figure 6:**
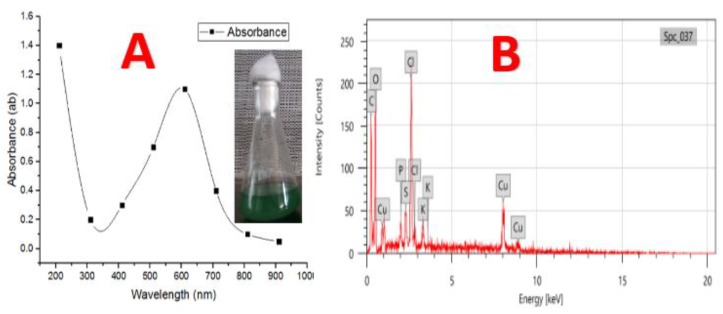
(**A**) UV–Vis spectrum of CuNPs. (**B**) Energy-dispersive X-ray spectroscopy (EDX) analyzer image of biotransformation of CuNPs by *P. fluorescens* MAL2.

When a cell-free extract of *Pseudomonas fluorescens* MAL2 was added to CuSO_4_·5H_2_O solution in the growing flasks and left for incubation for 48 h, the reaction mixtures color changed from blue to heavy green (Figure 5A), proving the biotransformation of CuNPs. The analogous appearance of a dark green color solution due to the addition of 5 mM CuSO_4_ to a flask comprising *Morganella* sp., indicated CuNPs biotransformation [47,48]. When different concentrations of CuSO_4_·5H_2_O solutions were added to the pellet, no color change occurred (Figure 5B). The UV–Vis absorption spectrum of this reaction mixture exhibited a specific absorption peak in the range of 550 to 650 nm (Figure 6A), which can be attributed to CuNPs [49]. The exact position of Surface Plasmon Resonance (SPR) band may shift, which depends on the properties of individual nanoparticles, including size, shape, and capping agents [50]. 

#### 4.5.2. EDX

EDX analysis proved that CuNPs were designed as aggregates and had mutable morphology (Figure 6B). The EDX analysis illustrated that 13.21% of copper and 22.55% of oxygen and another element were present in nanoparticles. The appearance of other elements may be due to a mixture of reaction components or other biomolecules secreted by the tested bacteria. Another element appears with the nanoparticles as per the medium components used in bacterial growth, which was also reported by Shantkriti and Rani [27]. The EDX results showed the presence of oxygen, carbon, phosphorus, chlorine, potassium, and sulfur elements in the purified bioflocculant. The presence of oxygen, copper, phosphorus, magnesium, calcium, chlorine, and sulfur elements in the as-synthesized nanoparticles represented the passivation of the copper nanoparticles with the bioflocculant [51]. The EDX results confirmed the presence of Cu in a sample which was an indication that the bioflocculant could be used as a capping agent in CuNPs biotransformation.

#### 4.5.3. Transmission Electron Microscope (TEM)

**Figure 7:**
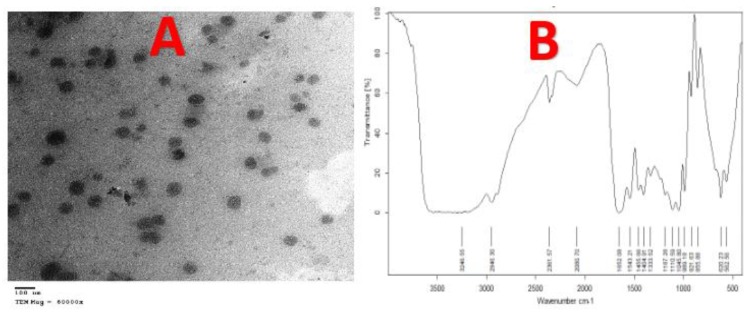
(**A**) TEM electron micrograph of the CuNPs. (**B**) FTIR analysis of the biotransformation CuNPs by *P. fluorescens* MAL2.

TEM was performed to analyze the shape and size of CuNPs [52]. Figure 7A revealed TEM images of CuNPs attained after 48 h of the reaction of cell-free extract (*P. fluorescens* MAL2) with CuSO_4_·5H_2_O; the CuNPs size was in range of 10 to 70 nm with the presence of spherical aspect. The size of CuNPs produced by *Pseudomonas fluorescens* ranged between 20 and 80 nm with the presence of spherical and hexagonal NPs. These results are in line with those reported by Shantkriti and Rani [27]. From the study of Dlamini et al. [51], the obtained TEM image showed that as-synthesized copper nanoparticles, using a bioflocculant extracted from *Alcaligenes faecalis* HCB2, were close to the spherical shape. The copper nanoparticles appeared to be close to spherical in shape and aggregation [51].

#### 4.5.4. Fourier Transform Infrared (FTIR) Analysis

This analysis gave data of the functional groups presented in compounds in the bacterial supernatant that interacted with metal ions. The FTIR spectrum of CuNPs synthesized using *Pseudomonas fluorescens* MAL2 supernatant is shown in Figure 7B. The presence of seventeen bands at 3246.56, 2946.30, 2361.57, 2080.70, 1652.08, 1543.21, 1455.66, 1404.91, 1333.52, 1187.28, 1110.59, 1045.80, 989.18, 921.62, 855.88, 620.23, and 562.56 cm^−1^ were recorded. These bands represented different functional groups such as primary and secondary amines, amides, alcohols, phenols, and strong and broad peak with the bonds of O–H stretching and N–H stretching at 3246.56 cm^−1^, whereas at 2946.30, 2361.57, and 2080.70 cm^−1^ gave strong and weak peaks, having functional groups of aromatics and alkynes, respectively. The bands appearing at 1652.08, 1455.66, 1404.91, and 1333.52 cm^−1^ were assigned for –C≡C– stretching and C–C stretching (in-ringing) of alkenes and aromatics, respectively. Moreover, a strong peak at 1543.21 cm^−1^ corresponded to N–O asymmetric stretching vibrations of nitro molecules. The crest of absorbance at 1187.28, 1110.59, and 1045.80 cm^−1^ shows C–O stretching of phenol and alcoholic compounds. The peaks at 989.18, 921.62, and 855.88 cm^−1^ are due to aromatic C–H bending. The peak at 620.23 cm^−1^ is as per aromatic C–H bending. The peak at 562.56 cm^−1^ is as per metal–carbon stretch. The recorded FTIR analysis suggests that the CuNPs might be surrounded by organic molecules such as polyphenols, alkaloids, and terpenoids, as already reported [53]. The carbonyl groups of the amino acids residues and some peptides have a strong ability to couple to the NPs [54]. Many scholars reported that the proteins could bind to NPs either through free amine or cysteine groups in proteins. These proteins that prevail over the NPs surface might act as a capping agent for stabilization [55].

### 4.6. Dynamic Light Scattering (DLS) Analysis

**Figure 8:**
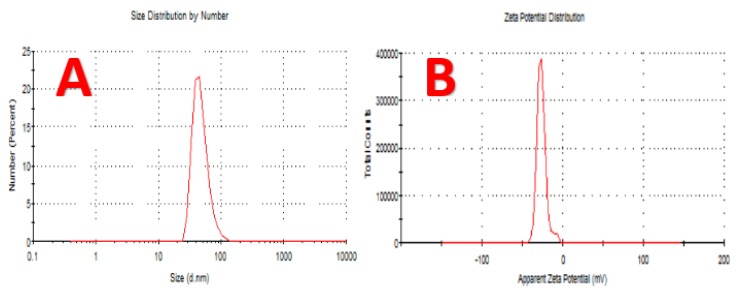
(**A**) The particle size distribution of biotransformation CuNPs. (**B**) Zeta potential of the biofabrication CuNPs by *P. fluorescens* MAL2.

The average size of the CuNPs, size distribution, and polydispersity index (PDI) of the biotransformation CuNPs were estimated by Dynamic Light Scattering (DLS), and the results are given in Figure 8A. The results show that the average CuNPs size was 48.07 nm and the polydispersity index was 0.227. The average NPs size and PDI showed that the biotransformed CuNPs were monodispersed.

### 4.7. Zeta Potential Analysis

To observe the surface charges acquired by CuNPs, Zeta potential analysis was conducted, which can be performed to have clear prevision into the stability of the formed colloidal CuNPs. The magnitude of zeta potential gives an insight into inherent stability of colloid. It should be observed that the particles of CuNPs, with zeta potential values which were more positive than +30 mV or more negative than −30 mV, are deemed to be stable [56]. The zeta potential of the CuNPs in this study was found to be −26.00 mV (Figure 8B), and it can be inferred that the biotransformed CuNPs are very stable [57]. The electrostatic repulsive force between the NPs depends on the charge that resides on the surface of nanoparticles. When the charge of the nanoparticles is negative, it does not cause the aggregation of these nanoparticles, causing long-term stability. 

### 4.8. Toxic Effect of Nano-Copper on Tribolium castaneum

Table 1 shows the toxic effect of Bio CuNPs against *T. castaneum* in comparison with Ch CuNPs. The mortality rate of *T. castaneum* adults increased with the increase in Bio CuNPs concentration concerning the exposure time. More specifically, the LC_50_ of Bio CuNPs after 24 h exposure of *T. castaneum* adults was 693.7 ppm. This value decreased dramatically to 130.5 and 36.89 ppm after three and five days, respectively. Besides, *T. castaneum* was suppressed at the higher concentration of Bio CuNPs (300 ppm) after 5 days of exposure. This effect may be attributed to the ability of nanoparticles to pass through the epithelial and endothelial cells by transcytosis [58]. Furthermore, nanoparticles can proceed easily along the dendrites, axons, blood, and lymphatic vessels, causing oxidative stress and other effects [59]. On the contrary, any mortality of *T. castaneum* adults was observed with Ch CuNPs at all used concentrations. Also, the control samples did not show any mortality. Therefore, it was obvious that the same concentration of Bio CuNPs and Ch CuNPs did not provide the same effect on *T. castaneum*. Two possible reasons could explain the negative effect of Ch CuNPs: First, the concentrations of Ch CuNPs were not adequate to kill *T. castaneum* and should be increased. This hypothesis was further supported by Shaker [60] who reported a mortality rate of 95% when cotton leafworm *Spodoptera littoralis* was exposed to chemically synthesized CuO-NPs at 1000 ppm. Secondly, Gudikandula [61] demonstrated that a stabilizer agent should be added to enhance the effect of Ch CuNPs and prevent agglomeration. 

On the other hand, the positive effect of the different chemical under nanosize as biocontrol against different pests has been reported in several investigations. For instance, the populations of two-spotted spider mites, the most critical bean pests, were significantly decreased with the addition of nano-capsule of CuO [62]. Selvan [63] found larvicidal activity against the transmit vector *Aedes aegypti* when treating with CuO-NPs using greenly synthesis using leaf extract of *Tridax procumbens*. Besides, it was proposed that the application of nanotechnology in agrochemical formulations can reduce the petrochemicals used in pesticides [64]. Recent studies demonstrated that the combination of AgNPs with a lesser quantity of Malathion produced high insecticidal efficacy for *T. castaneum* control [65]. Taking into consideration the positive effects of nanoparticles as biocontrol agents that have been confirmed by aforementioned studies together with the fact that the concentration of nanoparticles could be reduced with an effective mortality rate when they were produced biologically, it can be concluded that the biological production of nanoparticles could be considered an ecofriendly approach. This conclusion is also supported by Selvan and Gunalan, who demonstrated that both biogenic and green synthesis of Cu-NPs emphasized their insecticidal efficacy as an ecofriendly and inexpensive method [63,66]. Note that even if the current investigation demonstrated that nanoparticles, i.e., Cu-NPs, have the insecticidal potential against the stored product pests, with particular reference to *T. castaneum*, the same conclusion should not be assumed in other pests and each pest should be evaluated separately. Accurately, it was reported that larvae of *T. confusum* were more susceptible to the exposure of nano-Diatomaceous earth than *T. castaneum* larvae [67]. In addition, further investigations are still required for the utilization of high concentration of such nanoparticles. For instance, despite CuO-NPs showing the potential to eliminate alachlor and phenanthrene in aqueous solutions [68], the high concentration of CuO-NPs in aqueous solutions led to intense accumulation in the larval body of the freshwater shredder *Allogamus ligonifer*; where LC_50_ value was 569 mg L^−1^ after 96 h of exposure, resulting in negative effects on their growth and feeding behavior [69].

## 5. Conclusions

Green manufacturing of copper nanoparticles is a safe, cost-effective, and ecofriendly process against the most critical economic insects such as *Tribolium castaneum*, which cause significant losses in crops that enter human and animal food chains. The *Pseudomonas fluorescens* strain that was isolated in the present study showed competency in copper nanoparticle synthesis in sizes ranging from 10 to 70 nm. It could be concluded that the biologically synthesized of copper nanoparticles have an excellent insecticidal activity at low concentrations against the stored product pest *T. castaneum*. The effect of biologically synthesized of copper nanoparticles excelled that of chemically synthesized of copper nanoparticles.

## Figures and Tables

**Table 1:** Probit analysis for the data on adult mortality of *Tribolium castaneum* treated with CuNPs at 95% confidence limits.

Treatments		CuNPs Concentration (μg/mL)	300		250	200		150		100	50	LC50 (μg/mL)	Chi-square Value
		Period	%Mortality										
Bio CuNPs		First day	30		20	17		10		7	3	693.7	0.589
		Third day	67		60	50		47		43	43	130.5	1.67
		Fifth day	100		93	83		73		70	67	36.89	6.63
Ch CuNPs		-	-		-	-		-		-	-	-	-
Negative Control (distilled H_2_O)		-		-			-		-			-	-
Positive Control	*Pseudomonas* supernatant	-		-			-		-			-	-
	CuSO_4_·5H_2_O	-		-			-		-			-	-

LC_50_: lethal concentration that kills 50% of the exposed adults; Bio CuNPs: biological of Copper nanoparticles; Ch CuNPs: chemical synthesis of Copper nanoparticles.

## Abbreviations

FTIR: Fourier-transform infrared spectroscopy; LC50: lethal concentration 50; PDI: polydispersity index; Ch CuNPs: Chemical copper nanoparticles; DSM: Deutsche Sammlung von Mikroorganismen; CFU: Colony forming unit; Rh: Relative humidity; Cu^+2^: Copper ion.
